# Changing epidemiology of *Listeria monocytogenes* outbreaks, sporadic cases, and recalls globally: A review of ProMED reports from 1996 to 2018

**DOI:** 10.1016/j.ijid.2019.04.021

**Published:** 2019-04-30

**Authors:** Angel N. Desai, Amylee Anyoha, Lawrence C. Madoff, Britta Lassmann

**Affiliations:** a Brigham & Women’s Hospital, Division of Infectious Diseases, Boston, MA, USA; b International Society for Infectious Diseases, Boston, MA, USA; c Tufts University, Boston, MA, USA; d University of Massachusetts, Division of Infectious Diseases, Worcester, MA, USA

**Keywords:** *Listeria*, Food safety, Disease surveillance, Epidemiology

## Abstract

**Background:**

The purpose of this study was to identify global trends in *Listeria monocytogenes* epidemiology using ProMED reports. ProMED is a publicly available, global outbreak reporting system that uses both informal and formal sources. In the context of *Listeria*, ProMED reports on atypical findings such as higher than average case counts, events from unusual sources, and multinational outbreaks.

**Methods:**

Keywords “*Listeria*” and “listeriosis” were utilized in the ProMED search engine covering the years 1996–2018. Issue date, countries involved, source, suspected and confirmed case counts, and fatalities were extracted. Data unique to each event, including commentary by content experts, were evaluated. When multiple reports regarding the same outbreak or recall were obtained, the last report pertaining to that outbreak was utilized. Rates of *Listeria* events over time were compared using a normal approximation to the Poisson distribution; *p* < 0.05 was considered to be statistically significant.

**Results:**

From 1996 through 2018, 123 *Listeria* events were identified in the ProMED database. Eighty-one events (65%) were associated with two or more human cases (outbreak events), 13 events (11%) were associated with only one human case (sporadic cases), and 29 events (24%) were precautionary food product recalls due to the presence of bacterial contamination without associated human cases. The implicated food vehicle was identified in 69 (85%) outbreak events and in 10 (77%) sporadic case events. *Listeria* contaminated foods were identified in all precautionary recall events. Overall, 28 events (23%) implicated novel food vehicles/sources. Events associated with novel food vehicles increased over the study period (*p* < 0.02), as did international events with more than one country involved (*p* < 0.02). Ten reports (8%) described hospital-acquired events.

**Conclusions:**

This study demonstrates the use of publicly available data to document *Listeria* epidemiological trends, particularly in settings where foodborne disease surveillance is weak or nonexistent. Over the last decade, an increasing number of events have been associated with foods not traditionally recognized as vehicles for *Listeria* transmission, and a rise in international events was noted. Informing high-risk individuals such as pregnant women and immunocompromised individuals of safe food handling practices is warranted. To ensure timely recall of contaminated food products, open data sharing and communication across borders is critical. Changes in food production and distribution, and improved diagnostics may have contributed to the observed changes.

## Introduction

*Listeria monocytogenes* is a bacterial foodborne pathogen implicated in sporadic cases, outbreaks, and food recalls worldwide. Infection can be asymptomatic or cause a range of diseases from mild gastroenteritis in immunocompetent individuals to invasive disease in high-risk groups including the elderly, children, immunocompromised individuals, and pregnant women (Ferreira et al., 2014). Pregnancy-associated listeriosis can result in preterm birth, miscarriage, or stillbirth (Mylonakis et al., 2002; Lamont et al., 2011).

Recent large-scale events such as the outbreak that occurred in South Africa from 2017 to 2018 have garnered renewed interest in the potential for widespread disease. This may be facilitated by changes in food production and distribution processes (Allam et al., 2018). The outbreak in South Africa was the largest *Listeria* outbreak to date, with more than 1000 laboratory-confirmed cases and more than 200 fatalities (NICD, 2019). A ready-to-eat, processed meat product called polony was identified as the source using whole-genome sequencing (WGS). The outbreak strain was also identified in the manufacturer processing environment of the implicated product. Recalls were subsequently issued in South Africa and 15 other African countries to which the product was distributed (World Health Organization (WHO), 2019). In the USA, whole cantaloupe melons from a single farm were associated with a large, multistate outbreak in 2011 (*n* = 147 cases). This event caused the most deaths from an outbreak of foodborne illness in the USA in more than 80 years (*n* = 33 deaths) (Centers for Disease Control and Prevention, 2011).

Modern day food systems are diverse and complex. The global movement of foods, processing supplies, and ingredients, coupled with the ability of *Listeria* to persist and multiply, facilitate the spread of contaminated products across borders, often resulting in mass recalls. Food recalls are not only a public health issue, but can also cause significant economic losses. In the past, foods most commonly identified as vehicles of *L. monocytogenes* transmission included unpasteurized milk and dairy products, soft cheese varieties (such as queso fresco, feta, and camembert), cooked ready-to-eat sausages and sliced meats (often referred to as ‘deli meats’), refrigerated smoked seafood, and refrigerated pâtés or meat spreads. Over the last decade, several novel food vehicles have been implicated in listeriosis outbreaks and recalls, such as raw sprouts and the previously mentioned whole cantaloupe melons (European Food Safety Authority, 2009; Allerberger and Wagner, 2010; McCollum et al., 2013).

Although *L. monocytogenes* infection is a mandated notifiable disease in some countries, this is not the case in many others. As of 2016, data from European countries that collect information on *L. monocytogenes* infections reported 2555 confirmed listeriosis cases in the European Union (EU)/European Economic Area (EEA), with the highest rates detected in infants below 1 year of age (1.3 per 100 000 population) and among elderly people over 64 years of age(1.6 per 100 000) (Listeriosis, 2019). In France, which has a robust national surveillance system, 375 cases were reported in 2016 (Listeriosis, 2019). Similarly, the USA reported 675 confirmed cases in 2014 (Foodnet, 2019). Listeriosis is more often a sporadic illness, as was illustrated in a report from the Foodborne Diseases Active Surveillance Network (FoodNet) in the USA (Foodnet, 2019).

When national surveillance data are lacking, informal disease surveillance systems provide an opportunity to understand epidemiological trends, particularly in cross-border settings. ProMED, the Program for Monitoring Emerging Diseases, is based on informal disease surveillance, which allows it to gather information on a variety of emerging and re-emerging diseases and disseminate this information more rapidly than traditional surveillance systems (Brownstein et al., 2009; Carrion and Madoff, 2017). Using publicly available data to document trends in *Listeria* epidemiology may translate into valuable information for food safety enhancement, particularly in settings where foodborne disease surveillance is weak or non-existent. The purpose of this study was to better characterize global *L. monocytogenes* events through an analysis of ProMED reports over time.

## Methods

### Data source

ProMED, the Program for Monitoring Emerging Diseases, is an infectious disease surveillance network established in 1994. ProMED currentlyhosts50subjectmatterexpertslocatedin35countrieswho actively monitor media, professional networks, and other informal data sources to detect the earliest warning signs of an emerging infectious disease or toxic exposure event. ProMED followers often submit reports of cases or findings as well. The subject matter experts provide expert commentary, supply references to previous reports and scientific literature, and put reports in perspective for a diverse audience (ProMED (2019)). ProMED focuses primarily on the reporting of emerging and re-emerging outbreak events that are of global concern, unusual, or in excess of what would normally be expected in a particular region or country. It is used daily by public health leaders, government officials, physicians, veterinarians, researchers, private companies, journalists, and the general public. ProMED is independent, transparent, and committed to the open sharing of data. Meta-tagged information and full-text ProMED reports are stored in a central database with more than 55 000 individual records at present.

### Data extraction

The ProMED database search engine was queried for entries between January 1996 and December 2018 using keywords “*Listeria*” and “listeriosis”. Of the 55 409 records, 391 entries were initially retrieved and manually reviewed by three independent investigators. Duplicates and reports that did not address food-related events were removed. When multiple reports referring to the same event were extracted, only the latest report appearing in chronological order was included for final data analysis.

### Listeria event definitions

For the purpose of this study, an outbreak of listeriosis was defined as an event associated with two or more cases. Given the limitations of informal disease surveillance data, definitive linkage of isolates through molecular subtyping was not reported on ProMED for some outbreak events. A single case, even if demonstrably related to a particular *Listeria*-contaminated food, was classified as sporadic if no other cases were identified. Either of these categories may have been associated with food recalls. The term ‘implicated food vehicles’ refers to food vehicles potentially linked to clinical cases. Precautionary food recalls were defined as recalls of food contaminated with *Listeria* with no reported human cases. Collectively, outbreaks, sporadic cases, and precautionary recalls were labeled ‘events’.

### Descriptive analysis

Report date, countries involved, food source, suspected and confirmed case counts, case fatalities, and characteristics considered by the reviewers to be unique to the event were extracted for each entry. Events were further categorized as causing clinical disease with one or more cases versus food recall due to contamination. Microsoft Excel was used for the primary data collection and descriptive analysis.

### Novel food vehicles and international events

Foods most commonly identified as vehicles of *L. monocytogenes* transmission include unpasteurized milk and dairy products, soft cheese varieties (such as queso fresco, feta, and camembert), cooked ready-to-eat sausages and sliced meats (often referred to as ‘deli meats’), refrigerated smoked seafood, and refrigerated pâtés or meat spreads. For the purpose of this analysis, any additional food vehicles were defined as ‘novel’ items linked to *Listeria* transmission. International events were defined as involving two or more countries. In the case of precautionary recalls, contaminated food sources not traditionally associated with *Listeria* recalls were labeled ‘novel food sources’.

### Statistical analysis

Rates of multinational events per total number of *Listeria* events and rates of events linked to non-traditional food vehicles per total *Listeria* events reported in ProMED each year were calculated to ensure that trends were not due to changes in the total number of ProMED *Listeria* reports over time. The rates of *Listeria* events during different time periods were compared using a normal approximation to the Poisson distribution. Microsoft Excel and Stata (2013; StataCorp LP, College Station, TX, USA) were used for the statistical analysis. An α level of 0.05 was used and a *p*-value of <0.05 was considered to be statistically significant.

## Results

### Listeria event characteristics

**Figure 1.**
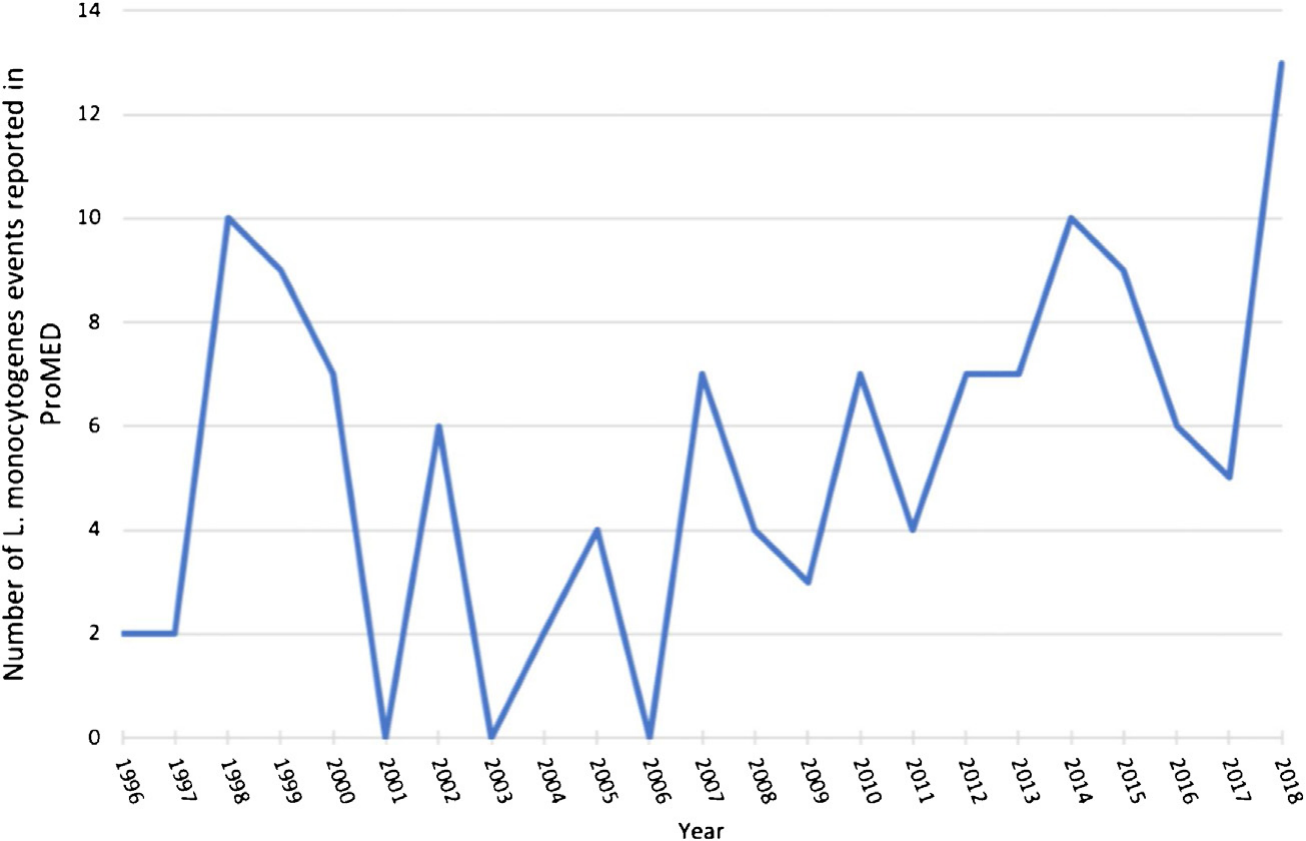
Occurrence of *Listeria monocytogenes* events reported in ProMED, per year.

One hundred and twenty-six *Listeria* events from 30 countries were identified in the ProMED database. Three reports described events involving domestic animals or pets and were excluded from further analysis. Study characteristics are summarized in Table 1. Of the remaining 123 events, 94 (76%) were associated with human cases, while 29 (24%) were categorized as a precautionary food recall due to the presence of bacterial contamination without associated human case(s). Of the 94 cases associated with human cases, 81 (65%) were associated with two or more human cases (outbreak events) and 13 events (11%) were associated with one human case (sporadic events). Figure 1 demonstrates the number of *Listeria*-associated events reported in ProMED per year. Case-fatality rates (CFR) associated with *Listeria* infection from ProMED reports were 20%. Although this is similar to the 20–30% worldwide CFR for listeriosis estimated by the US Centers for Disease Control and Prevention, it is likely an underestimate, as the last ProMED post for some outbreaks may not have reported final outcome data (US CDCP, 2019).

### Implicated food vehicles and contaminated foods

**Figure 2.**
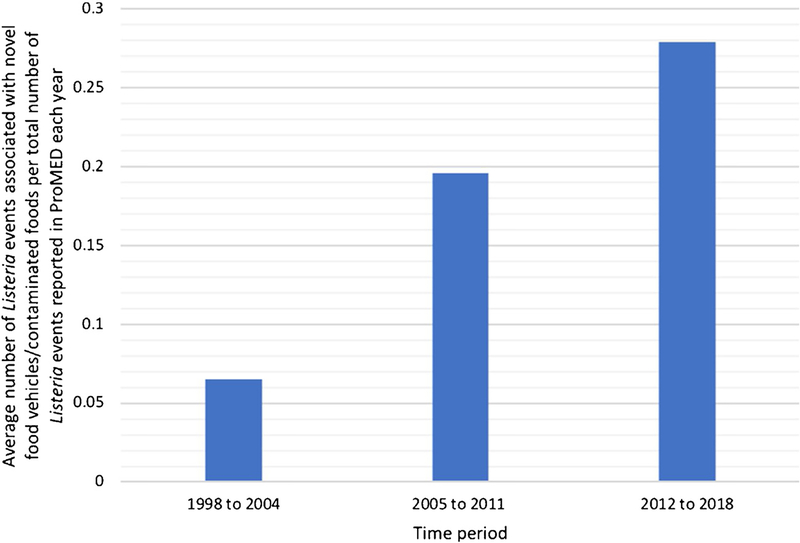
Average number of *Listeria* events associated with novel food vehicles and contaminated foods per total number of *Listeria* events reported in ProMED each year over three time periods.

Food vehicles were identified in 69 *Listeria* outbreak events (85%) and in 10 sporadic case events (77%) reported in ProMED. *Listeria* contaminated foods were identified in all precautionary recalls. Of note, 28 events (23%) implicated novel food vehicles or identied contaminated foods not traditionally recognized in precautionary recalls (Table 1). Table 2 demonstrates a list of novel food vehicles and contaminated foods linked to *Listeria* events identified in the ProMED database. For example, listeriosis outbreaks involving fruits such as cantaloupe melons, stone fruits (e.g., peaches, apricots), and caramel apples were reported, as were outbreaks related to desserts such as ice cream and profiteroles. The number of *Listeria* events associated with novel food vehicles or contaminated foods increased over time (Table 3). Rates of *Listeria* events linked to novel food vehicles per total number of *Listeria* events reported in ProMED each year were calculated and compared over three time periods, starting in 1998. Rates increased from 0.06 (1998–2004) to 0.28 (2012–2018), an overall increase of 366% (*p* < 0.02) (Figure 2).

### International events

**Figure 3.**
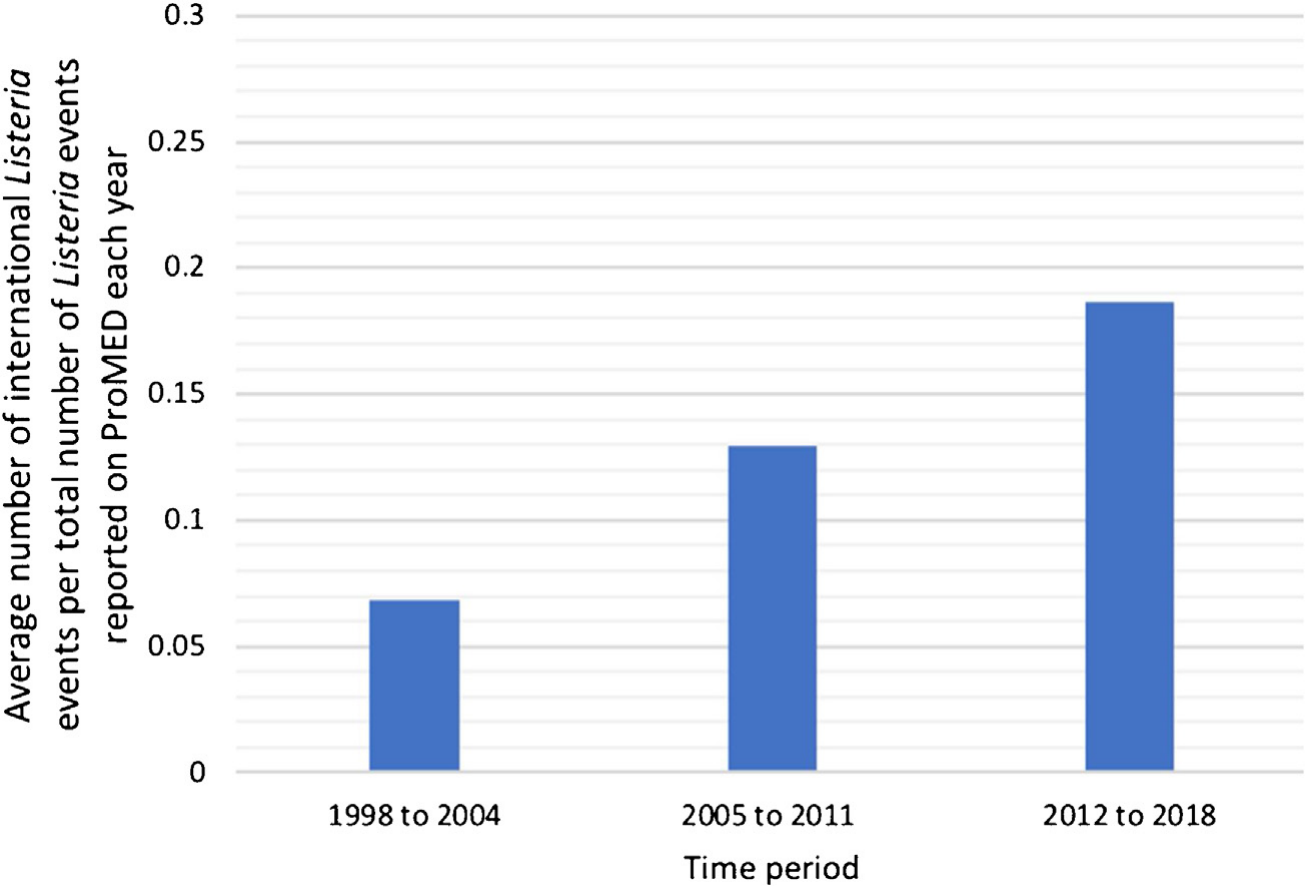
Average number of international *Listeria* events per total number of *Listeria* events reported in ProMED each year over three time periods.

Twenty-one (17%) of all events were noted to involve two or more countries. Of these, 17 events (81%) occurred over the last decade (2008–2018) as compared to only four reports (19%) occurring between 1996 and 2007 (Table 4). Rates of *Listeria* events involving two or more countries per total number of *Listeria* events reported in ProMED each year were calculated to ensure that any trends observed were not secondary to changes in the number of *Listeria* events reported. Figure 3 demonstrates the increasing rates of multi-country events from 1998 to 2018… No multi-country events were reported in 1996 and 1997. Rates increased from 0.07 (1998–2004) to 0.19 (2012–2018), an overall increase of 171% (*p* <0.02). Several of these events involved neighboring countries such as Canada and the USA, Austria and Germany, and Spain and Portugal. Other events involved countries in more than one continent. In 2018, *Listeria* was linked to cantaloupe melons from Australia that were distributed to eight countries including China (Hong Kong), Japan, Kuwait, Malaysia, Oman, Singapore, and the USA. In 2009, an outbreak with seven confirmed deaths was linked to chicken wraps distributed to airlines affecting approximately 5000 national and international flights.

### Hospital-associated events

Ten events (8%) were attributed to foods prepared and served in hospitals. Nine of these events were outbreaks with two or more associated human cases. One event was categorized as sporadic with only one reported human case. An outbreak that involved 13 public hospitals in Australia in 2013 was linked to proteroles while one in the USA in 2015 was linked to ice cream. Additional hospital-acquired cases were reported from Norway, the UK, and New Zealand.

### Recalls

Identification of the implicated food vehicle in *Listeria* outbreaks and sporadic cases resulted in numerous product recalls, some of which included products sold under different brands and distributed to multiple countries. During one outbreak in 2014, more than 358 consumer products sold under 42 different brands were recalled, including broccoli, cauliflower, peas, onions, peppers, sweet potatoes, blueberries, cranberries, peaches, raspberries, strawberries and others. The recent outbreak in South Africa related to contaminated polony resulted in recalls from 15 countries. Twenty-four percent of *Listeria* events (*n* = 29) reported in ProMED were precautionary food product recalls due to the presence of bacterial contamination without associated cases. For example, one early report in 1998 described a large recall of more than 35 million pounds (^~^16 million kilograms) of hot dogs and deli meats in the USA. Similarly in 2002, a recall in the USA involved 27.4 million pounds (^~^12.4 million kg) of deli meats, turkey, and poultry.

## Discussion

*Listeria monocytogenes* remains a potent source of foodborne infection worldwide. This study aimed to identify epidemiological trends in *Listeria* events and recalls since 1996. Relevant data were extracted from ProMED in order to elucidate global trends given the increasingly interconnected nature of food distribution practices worldwide. The past 20 years have witnessed an increase in *L. monocytogenes* events reported to ProMED. Many of those events have involved food vehicles that have not previously been implicated in *L. monocytogenes* transmission. Prior studies have argued that while improved control measures in some regions have decreased the prevalence of *L. monocytogenes*in food products such as meat and poultry, the rates of illness and particularly of invasive disease have remained steady or, in some cases, have increased over time, challenging previously held assumptions about the pathogen (Buchanan et al., 2017).

The reasons for increased reporting of events linked to non-traditional food vehicles noted in this study may be multifactorial. Cairns and Payne have posited that evolving food distribution practices and government regulations may be a contributing factor (Cairns and Payne, 2009). Ercsey-Ravaz et al. also noted that the global scale and complexities of modern food systems contribute to the likelihood and magnitude of foodborne illness (Ercsey-Ravasz et al., 2012). The prevalence and widespread consumption of ready-to-eat products may also play a role (Keto-Timonen et al., 2007; CDCP, 2011; Eurosurveillance Editorial Team, 2012).

In the past, the organism has often been difficult to enumerate due to limitations in laboratory detection, as well as a long incubation period (Auvolat and Besse, 2016). In addition, *L. monocytogenes*has the ability to form biofilms, grow despite refrigeration, and can be resistant to disinfectants (Keto-Timonen et al., 2007; Lundén et al., 2003). Diagnostic advances leading to early detection in recent years may be another factor associated with the changing epidemiology described in this study. While pulsed-field gel electrophoresis (PFGE) is often employed as an initial screening tool, the advent of reliable sequencing technology has allowed for more rapid outbreak identification (Datta and Burall, 2018). WGS, as used in South Africa and in other recent outbreaks, has allowed the improved identification of genetically related isolates (Allam et al., 2018). This in turn has assisted in establishing the presence of foodborne outbreaks. Within the EU/ EEA, listeriosis is one of the priority diseases for which supranational WGS-enhanced surveillance was initiated in 2018 (Van Walle et al., 2018). We expect that in the future, additional novel food vehicles will be linked to *L. monocytogenes* transmission. In addition, with increased compliance testing and more stringent regulatory frameworks for food safety at production and retail in many countries, food recalls are expected to increase accordingly – not only due to *Listeria*, but other foodborne pathogens as well.

It is noteworthy that several *Listeria* reports in the ProMED database related to foods prepared and served in hospitals. This is of concern, as a growing number of hospitalized patients are immunocompromised hosts susceptible to infectious diseases. The types of food products served in healthcare settings should be selected and prepared to minimize the risk of foodborne disease in vulnerable populations. The incorporation of hazard analysis and critical control points (HACCP) principles at every stage of food handling can be implemented to ensure food safety (FDA and HACCP, 2017). As cases may result from a breakdown in only one step of the production and distribution process, food processors, manufacturers, wholesalers, and retail outlets play a key role in maintaining the safety of food products and ingredients.

This appears to be the first global overview of listeriosis using an informal-source surveillance methodology. The strengths of this study lie in the use of ProMED data, which provide a snapshot of emerging infectious disease trends that can otherwise be difficult to elucidate. In many countries, listeriosis is not a notifiable disease, which can make routine data collection challenging. Given these limitations, informal-source surveillance provides an adjunct tool to characterize foodborne outbreaks. In addition, ProMED and other informal surveillance programs allow multinational reporting and comparison. This study found that 14% of all *Listeria*-associated events reported in ProMED involved multiple countries, highlighting the importance of cross-border food distribution surveillance and intense collaboration of public health and food safety authorities and laboratories on an international level.

While ProMED data may not provide a comprehensive review of all *Listeria* outbreaks, sporadic cases, or recalls, reporting on a global level over a long period of time allowed some conclusions to be drawn on *Listeria* epidemiology. This study was limited by the search terminology used in the database, which may have missed posts due to language choice. In addition, despite standardizing all reports by year, there is the possibility that increased reporting over the study time period in general may have contributed to some of the increases noted in *Listeria*-associated posts.

*Listeria monocytogenes* is an increasingly important pathogen to recognize given its high case-fatality rate compared to other foodborne diseases. Immunocompromised and other vulnerable individuals are among those at risk of serious disease sequelae. The use of advanced diagnostic technologies such as WGS, enforcement of food safety regulations, and raising awareness of the disease among high-risk groups are critical to infection and prevention control measures. Open data sharing using resources such as ProMED is equally important for supporting cross-border and multinational emerging disease surveillance.

## Figures and Tables

**Table 1:** Characteristics of *Listeria monocytogenes* events reported in the ProMED database.

Events	*n*, event counts (%)
Total events	123 (100%)
Outbreaks (two or more human cases)	81 (65%)
Sporadic cases	13 (11%)
Precautionary recalls^a^	29 (24%)
Hospital-acquired events	10/123 (8%)
Events involving multiple countries	21/123 (17%)
Case-fatality rate, overall	487/2383 (20%)

^a^ Precautionary recalls only without associated human case(s).

**Table 2:** Novel food vehicles and contaminated foods identified by ProMED Reports.

Food vehicles traditionally linked to *Listeria monocytogenes* transmission
Unpasteurized milk and dairy products Soft cheese varieties Cooked, ready-to-eat sausages and sliced meats (deli meats) Refrigerated seafood, smoked seafood Pâtés and meat spreads
Novel food vehicles linked to *Listeria monocytogenes* transmission reported in ProMED 1996–2018
Cantaloupe melons (whole and pre-cut), stone fruits (nectarines, peaches, plums, pluots) Asparagus soup Frozen fruits and vegetables including broccoli, cauliflower, peas, onions, peppers, sweet potatoes, corn, blueberries, cranberries, peaches, raspberries, strawberries and others Mashed potatoes Ice cream, profiteroles, caramel apples Sprouts Chopped celery, packaged salad mixes Sandwiches and wraps
Novel contaminated foods implicated in precautionary recalls not linked to human cases in ProMED 1996–2018
Sandwiches, burritos and bakery products Hummus and tahini Frozen french fries, ready to eat cheeseburgers Chopped romaine lettuce, alfalfa sprouts, crunchy sprouts, gourmet sprouts, spicy sprouts, radish sprouts, mustard-onion sprouts, dill sprouts and clover sprouts Sliced apples

**Table 3:** Number of *Listeria* outbreaks and sporadic cases associated with novel food vehicles and number of precautionary recalls associated with novel foods reported on ProMED over time.

	Outbreaks *n* = 81	Sporadic cases *n* = 13	Precautionary recalls *n* = 29
1996–2004	0	1	5
2005–2011	4	1	1
2012–2018	14	0	2

**Table 4:** Number of *Listeria* events involving two or more countries reported on ProMED over time.

	Outbreaks *n* = 81	Sporadic cases *n* = 13	Precautionary recalls *n* = 29
1996–2004	0	1	3
2005–2011	3	0	2
2012–2018	10	1	2
